# Insulin-Like Growth Factor-II and Ischemic Stroke—A Prospective Observational Study

**DOI:** 10.3390/life11060499

**Published:** 2021-05-29

**Authors:** Daniel Åberg, N. David Åberg, Katarina Jood, Petra Redfors, Christian Blomstrand, Jörgen Isgaard, Christina Jern, Johan Svensson

**Affiliations:** 1 Institute of Medicine, Department of Internal Medicine and Clinical Nutrition, The Sahlgrenska Academy, University of Gothenburg, 405 30 Gothenburg, Sweden; david.aberg@medic.gu.se (N.D.Å.); jorgen.isgaard@medic.gu.se (J.I.); johan.svensson@medic.gu.se (J.S.); 2 Region Västra Götaland, Sahlgrenska University Hospital, 413 45 Gothenburg, Sweden; katarina.jood@neuro.gu.se (K.J.); petra.redfors@vgregion.se (P.R.); christian.blomstrand@neuro.gu.se (C.B.); 3 Institute of Neuroscience and Physiology, Center of Brain Repair and Rehabilitation, The Sahlgrenska Academy, University of Gothenburg, 405 30 Gothenburg, Sweden; 4 Institute of Neuroscience and Physiology, Department of Clinical Neuroscience, The Sahlgrenska Academy, University of Gothenburg, 405 30 Gothenburg, Sweden; christina.jern@neuro.gu.se; 5 Institute of Biomedicine, Department of Laboratory Medicine, The Sahlgrenska Academy, University of Gothenburg, 405 30 Gothenburg, Sweden

**Keywords:** insulin-like growth factor-II, ischemic stroke, functional outcome, modified Rankin Scale, mortality, National Institutes of Health Stroke Scale (NIHSS)

## Abstract

Insulin-like growth factor-II (IGF-II) regulates prenatal brain development, but the role in adult brain function and injury is unclear. Here, we determined whether serum levels of IGF-II (s-IGF-II) are associated with mortality and functional outcome after ischemic stroke (IS). The study population comprised ischemic stroke cases (n = 492) and controls (n = 514) from the Sahlgrenska Academy Study on Ischemic Stroke (SAHLSIS). Functional outcome was evaluated after 3 months and 2 years using the modified Rankin Scale (mRS), and additionally, survival was followed at a minimum of 7 years or until death. S-IGF-II levels were higher in IS cases both in the acute phase and at 3-month follow-up compared to controls (*p* < 0.05 and *p* < 0.01, respectively). The lowest quintile of acute s-IGF-II was, compared to the four higher quintiles, associated with an increased risk of post-stroke mortality (median follow-up 10.6 years, crude hazard ratio (HR) 2.34, 95% confidence interval (CI) 1.56–3.49, and fully adjusted HR 1.64, 95% CI 1.02–2.61). In contrast, crude associations with poor functional outcome (mRS 3–6) lost significance after full adjustment for covariates. In conclusion, s-IGF-II was higher in IS cases than in controls, and low acute s-IGF-II was an independent risk marker of increased mortality.

## 1. Introduction

Insulin-like growth factor-I (IGF-I) and IGF-II promote growth, metabolism, regeneration, and repair mechanisms in the peripheral tissues and in the central nervous system (CNS) [1,2]. IGF-I and IGF-II are mainly produced in the liver [3], but there is also production in other tissues including the CNS, most prominently in neurons [4,5]. Furthermore, IGF-I as well as IGF-II can pass through the blood–brain barrier [6], and both the IGF-I receptor (IGF1R) and the IGF-II receptor (IGF2R) are widely distributed in the brain, predominantly in the brain endothelium and in the choroid plexus [7,8]. The IGF2R, which is also a mannose 6-phosphate receptor, has a high affinity for IGF-II, and a low affinity for IGF-I [7,8]. The IGF2R targets acid hydrolases and IGF-II to lysosomes [8,9]. However, the IGF2R can also recruit G proteins, thereby activating a cascade involving protein kinase C (PKC) and phospholipase C [8,10], which is distinct from that of the typical IGF-I signaling.

IGF-II is important for cell genesis and prenatal growth. In children, IGF-II mutations were associated with retarded longitudinal growth as well as speech and motor delay despite a compensatory increase in serum IGF-I [11]. In adulthood, the role of IGF-II is not clear, although it is several-fold more abundant than IGF-I in serum, and the level of s-IGF-II is less dependent on growth hormone (GH) than that of IGF-I [1,3,12]. However, experimental data support that IGF-II regulates CNS functions also in adult life. In rats, administration of IGF-II enhanced memory consolidation, although the effect was dependent on the exact timing, i.e., within a sensitive period, of IGF-II administration [13]. IGF-II was also involved in the effects on memory consolidation exerted by SHARP1 and SHARP2, two transcription factors regulating sleep/wake homeostasis [14]. In addition, normalization of IGF-II in aged rats improved memory functions [15], and IGF-II treatment provided protection from oxidative damage both in the aging rat brain [16] and after glucocorticoid exposure [17,18]. Interestingly, IGF-II might also affect the life span. A mouse model mimicking the human Silver–Russell syndrome was characterized by undetectable *Igf2* expression, fetal growth retardation, and perinatal lethality [19]. Human studies of the APAI polymorphism in the *IGF2* gene have shown that the APAI A allele is associated with higher serum IGF-II (s-IGF-II) and less obesity [16,20,21] as well as a prolonged life expectancy [22]. Finally, in Italian centenarians, there was an association in men between longevity and tyrosine hydroxylase–*IGF2* haplotypes on the 11p15.5 chromosomal region [23].

IGF-II could also be involved in the recovery from stroke, possibly by modulating the immunoreactive response of glial cells [24]. Furthermore, IGF-II was expressed in activated macrophages around an ischemic event [25], and the IGF2R was upregulated in ischemic neocortical pyramid neurons after focal cerebral ischemia in young hypertensive male rats [26]. Additionally, it has been possible to modulate IGF-II activity as the small protein RNA-binding motif protein 3 (RBM3) promoted IGF-II expression and secretion in the subgranular zone of the adult rodent dentate gyrus, thereby providing neuroprotection and neurogenesis after hypoxic–ischemic brain injury [27]. One human nested case–control study showed lower IGF-II levels in subjects with a subsequent ischemic stroke during follow-up compared to controls, although without statistical significance [28]. Otherwise, little is known of the role of IGF-II in human brain diseases. Studies on Alzheimer’s disease (AD) show conflicting results; serum IGF-II has been reported to be reduced [12], unchanged [29], or increased [30] in AD, whereas IGF-II in the cerebrospinal fluid (CSF) has mostly been observed to be increased [12,29].

In summary, recent data suggest that IGF-II is associated with life expectancy and CNS functions, and some experimental findings indicate protective effects of IGF-II in the ischemic brain. However, there is little knowledge of IGF-II levels in human ischemic stroke (IS). We therefore analyzed s-IGF-II in the Sahlgrenska Academy Study on Ischemic Stroke (SAHLSIS) and investigated associations with mortality and long-term functional outcome after IS.

## 2. Materials and Methods

### 2.1. Study Population

The design of SAHLSIS has previously been reported in detail [31,32]. Briefly, 600 patients with first-ever or recurrent acute IS before the age of 70 years were consecutively recruited at four stroke units in western Sweden. The controls were without a clinically known cardiovascular disease (N = 600) and randomly selected either from a population-based health survey or the Swedish Population Register, to match the patients in terms of age (<1 year), sex, and area of residence. This study was approved by the Ethics Committee of the University of Gothenburg. All participants or their next of kin provided written informed consent.

### 2.2. Vascular Risk Factors, Stroke Severity, and Subtypes

Body mass index (BMI), hypertension, diabetes mellitus, and smoking were registered as described previously [31,32]. Initial stroke severity was assessed in the acute phase (the maximum score during days 0–10) and after 3 months using the Scandinavian Stroke Scale (SSS). The SSS is similar to the more frequently used National Institutes of Health Stroke Scale (NIHSS) [33], although the scales are inverse (i.e., higher values indicate mild stroke in SSS). Therefore, the SSS scores were recalculated to NIHSS scores using the validated algorithm NIHSS = 25.68 − 0.43 × SSS [34]. Etiological subtypes were classified using the Trial of Org 10172 in Acute Stroke Treatment (TOAST) criteria [35] into the subtypes large vessel disease (LVD), small vessel disease (SVD), cardioembolic (CE) stroke, cryptogenic stroke (when no cause was identified despite extensive evaluation), other determined cause of stroke, and undetermined stroke using a local specified protocol as described previously [31].

### 2.3. Functional Outcome and Mortality

Functional outcome was evaluated after 3 months and 2 years using the modified Rankin Scale (mRS). We dichotomized the mRS score for poor outcome (mRS 3–6), indicating death or functional dependence, vs. good outcome (mRS 0–2), indicating functional independence. We used the unique 10-digit Swedish person identity number to identify all patients who had died from the Swedish Population Register (Folkbokföringen). The follow-up time was a minimum of 7 years or until death.

### 2.4. Blood Sampling and Biochemical Analysis

Venous blood samples were collected in the acute phase (median 4 days, range 1–10 days after the index stroke) and at the 3-month follow-up (median 101, range 85–125 days) in IS cases and once in controls. Blood sampling was performed between 08:30 and 10:30 a.m. after an overnight fast of > 8 h. Serum was isolated within 2 h by centrifugation at 2000× *g* at 4 °C for 20 min and stored at −80 °C for before assay. S-IGF-II levels were measured by an enzyme linked immunosorbent assay (ELISA) using a commercial kit from Mediagnost, Reutlingen, Germany. The inter-assay coefficient of variation was 7.0%, and the intra-assay coefficient of variation was 4.2%. All blood and plasma concentrations of glucose and low-density lipoprotein cholesterol (hereafter LDL) were analyzed using standardized methods at the Department of Clinical Chemistry at the Sahlgrenska University Hospital. HOMA-IR was calculated as fasting insulin (microU/L) × fasting glucose (nmol/L)/22.5 [36]. High-sensitivity CRP (hs-CRP) was analyzed in serum by a solid-phase chemiluminescent immunometric assay on Immulite 2000 (Diagnostic Products Corp, Los Angeles, CA, USA) with the manufacturer’s reagents as directed [37].

### 2.5. Statistical Evaluation

The statistical analyses were performed using SPSS version 26.0 (IBM Corp., Armonk, NY, USA). Serum IGF-II was normally distributed according to the Kolmogorov–Smirnov test. Between-group differences were analyzed using ANOVA followed by Tukey’s HSD post hoc test for continuous variables and using chi-square tests for categorical variables. Correlation coefficients were calculated using Pearson correlation analysis.

Kaplan–Meier survival curves were constructed to exhibit survival rates in individual quintiles of s-IGF-II as well as in the lowest quintile of s-IGF-II compared to the merged group of the four higher s-IGF-II quintiles. In the following regression analyses, we compared the lowest quintile of s-IGF-II with that in the merged group of the four higher s-IGF-II quintiles. This is in line with earlier studies of biological and endocrine biomarkers and the IGF family, which implicate an inverse nonlinear excess risk in deficient subjects and that there may be a threshold level of IGF-II [28,38,39]. We evaluated whether s-IGF-II was associated with all-cause mortality (henceforth mortality) by calculating hazard ratios (HRs) and 95% confidence intervals (CIs) using Cox proportional hazards regression analysis comparing the lowest quintile of s-IGF-II with that in the merged group of the four higher s-IGF-II quintiles. Furthermore, to evaluate whether s-IGF-II was associated with poor functional outcome (mRS 3–6), using binary logistic regression analysis, we calculated odds ratios (ORs) and 95% CIs comparing the risk in the lowest quintile of s-IGF-II with that in the merged group of the four higher s-IGF-II quintiles.

To examine the independent effect of IGF-II on mortality and functional outcome, in line with that performed previously [32,40], adjustments were made for covariates related to stroke severity and well-known metabolic and cardiovascular risk factors. In these analyses, as LDL had the most missing values, imputation was used to replace the missing values with the mean LDL value. A two-tailed *p*-value of < 0.05 was considered statistically significant.

### 2.6. Ethics Approval and Consent to Participate

This study was conducted in accordance with the 1964 Helsinki Declaration and its later amendments or comparable ethical standards. Participants or next of kin provided written informed consent. This study was approved by the Ethics Committee of the University of Gothenburg (Ö469-99 and T553-03). The Ethics Committee of the University of Gothenburg is responsible for ethical applications from the entire region of western Sweden, covering all four hospitals in the study.

## 3. Results

### 3.1. Baseline Characteristics

In the present study, we included 492 patients and 514 controls that had successful analysis of IGF-II, with clinical characteristics provided in Table 1. The mean age at inclusion was 57.2 years, and 64% were men. As previously reported [31,32,40], IS cases had more hypertension, diabetes mellitus, smoking, and higher HOMA-IR as well as hsCRP than matched healthy controls. However, LDL levels and BMI did not differ significantly. Although serum IGF-II was higher in IS cases both in the acute phase and after 3 months compared to controls (*p* < 0.05 and *p* < 0.01, respectively), there was no significant post-stroke difference between the acute and 3-month s-IGF-II levels. With regard to IS subtypes, both acutely and after 3 months, s-IGF-II was significantly higher in cryptogenic stroke (7.3%), and lower in cardioembolic stroke (6.2%), as compared with the controls (Table 1).

**Figure 1:**
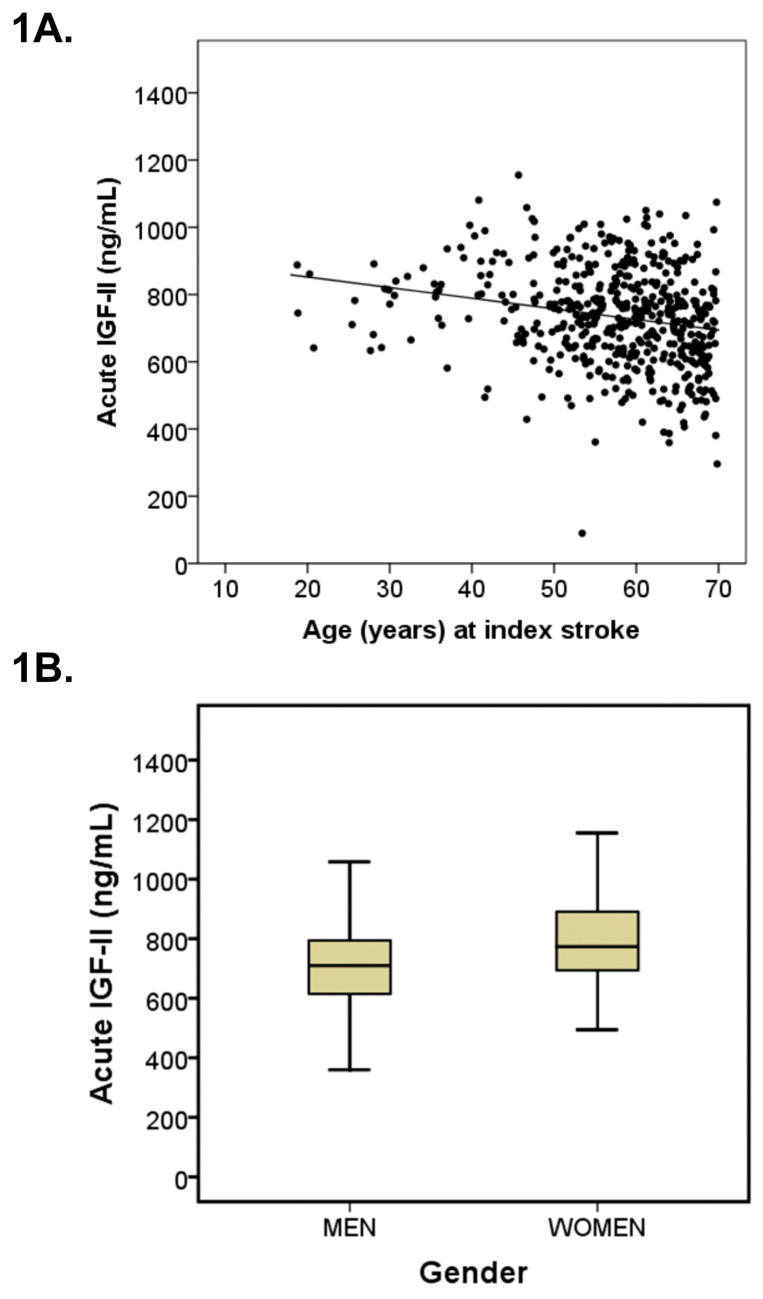
S-IGF-II and age and sex in the acute phase of ischemic stroke. (**A**) S-IGF-II levels in the acute phase were inversely correlated with age in the total stroke cohort (n = 492; r = −0.20, *p* < 0.001). The equation for the relation is s-IGF-II (ng/mL) = 916 − [3.16 × age (years)], valid through 18 to 70 years of age. (**B**) Boxplots of s-IGF-II in men (n = 315) and women (n = 177) in the acute phase after stroke. Values in the box plots are provided as medians (horizontal lines), 25–75th percentiles (boxes), and ranges (whiskers). Stroke men had lower acute s-IGF-II than stroke women (*p* < 0.001).

Serum IGF-II was lower in older subjects as reflected by inverse correlations between age and s-IGF-II (acutely: r = −0.20, *p* < 0.001 (Figure 1A); after 3 months: r = −0.13, *p* < 0.001; and in controls: r = −0.10, *p* < 0.001). Moreover, acute but not 3-month s-IGF-II correlated inversely with NIHSS, i.e., stroke severity (r = −0.10, *p* = 0.03 and r = 0.002, *p* = 0.95, respectively; not shown). Furthermore, acute s-IGF-II correlated negatively with hsCRP (r = −0.24, *p* < 0.001), and positively with LDL (r = 0.14, *p* = 0.002), but not with day of blood sampling (r = 0.02, *p* = 0.72), insulin resistance (HOMA-IR; r = −0.04, *p* = 0.43), or BMI (r = −0.03, *p* = 0.57).

Women had higher values of s-IGF-II compared to men. S-IGF-II of women in the acute phase of stroke was 10.7 % higher (*p* < 0.001, Figure 1B) and 11.6% higher 3 months after stroke (*p* < 0.001), and 7.7 % higher among the controls (*p* < 0.001).

### 3.2. S-IGF-II and Risk of Mortality

**Figure 2:**
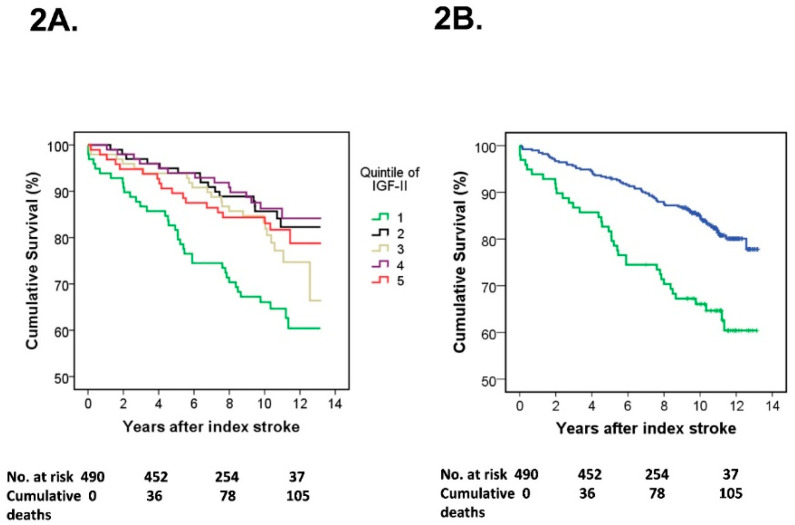
Low s-IGF-II in the acute phase of ischemic stroke (IS) is associated with increased risk of mortality. Kaplan–Meier survival curves for the risk of all-cause mortality are shown for (**A**) all individual quintiles of acute s-IGF-II in the total IS cohort, and (**B**) the lowest quintile of acute s-IGF-II (quintile 1) and the merged group of the four higher acute s-IGF-II quintiles (quintiles 2–5) in the total IS cohort. Vertical markers within the lines represent censored data. In (**A**), the individual quintiles of acute s-IGF-II are shown in the colors specified in the panel. In (**B**), quintile 1 of acute s-IGF-II is displayed as a green line and the merged quintiles 2–5 of acute s-IGF-II as a blue line. Numbers at risk and cumulative mortality are presented at the index stroke and after 4, 8, and 12 years post-stroke. Panel A: log-rank tests: *p* = 0.001 quintile 1 vs. quintile 2; *p* = 0.025 quintile 1 vs. quintile 3; *p* < 0.001 quintile 1 vs. quintile 4; and *p* = 0.06 quintile 1 vs. quintile 5. Panel B: log-rank test: *p* < 0.001 quintile 1 vs. the merged quintiles 2–5.

The follow-up time was a minimum of 7 years or until death (median 10.6, interquartile range 9.5–11.6 years). Kaplan–Meier survival curves showed an increased rate of post-stroke mortality in the lowest quintile of acute s-IGF-II compared to that in the other quintiles (Figure 2A). In contrast, the mortality rate was approximately similar in quintiles 2–5 of acute s-IGF-II, providing the rationale for merging these (as described in more detail in the Methods section). Accordingly, when mortality was assessed as a comparison between quintile 1 vs. quintiles 2–5, low acute s-IGF-II was still associated with increased mortality (log-rank test, *p* < 0.001, Figure 2B). Furthermore, in Cox proportional hazards regression analyses using the merged four highest IGF-II quintiles as reference, low acute s-IGF-II was associated with increased mortality (quintile 1 vs. quintiles 2–5: crude HR 2.34, 95% CI: 1.56–3.49, fully adjusted (model D): HR 1.64, 95% CI: 1.02–2.61; Table 2). In contrast, s-IGF-II quintiles after 3 months were not associated with mortality (data not shown).

### 3.3. Serum IGF-II and Functional Outcome

In the total cohort (n = 490), binary logistic regression analyses showed that the lowest quintile of acute s-IGF-II was associated with increased risk of poor functional outcome (mRS 3–6) after 3 months (crude OR 2.30, 95% CI 1.39–3.82) and 2 years (crude OR 1.93, 95% CI 1.17–3.18). These ORs remained statistically significant with adjustments for age and sex, attenuated after adjustment for cardiovascular covariates, whereas the associations were not retained after adjustments for stroke severity and hsCRP (Table 3).

S-IGF-II quintiles after 3 months were not associated with functional outcome after 3 months or 2 years (data not shown).

## 4. Discussion

Previously, serum IGF-I and insulin resistance have been associated with IS outcome [32,40,41,42]. Here, we investigated the importance of IGF-II, which is more abundant in serum than IGF-I in adult life [1,12]. We found that both in the acute phase and after 3 months, s-IGF-II levels were modestly higher in IS cases than in the controls. The lowest quintile of acute s-IGF-II was associated with increased mortality also after full adjustment for covariates. In contrast, the associations between low acute s-IGF-II and poor functional outcome after 3 months and 2 years were not retained after full adjustments for covariates.

### 4.1. IGF-II Levels in Normal Subjects and Stroke Patients

The role of IGF-II in fetal life is well established, but there is a paucity of IGF-II studies in adult human life. There are a few studies that have reported that s-IGF-II slowly declined with increasing age in healthy male subjects [43]. Additionally, among healthy elderly subjects (≥60 years), s-IGF-II tended to be lower in men compared to women [44]. Here, we confirm that s-IGF-II is inversely associated with age in healthy subjects, and that s-IGF-II is lower in men than in women. We extend the previous knowledge by showing that also in subjects with IS, there is a negative association between s-IGF-II and age as well as a sex difference in s-IGF-II. We also found modestly elevated s-IGF-II levels in IS cases, both in the acute phase (3.2% higher than in controls) and after 3 months (3.5%). In a study by Johnsen et al., investigating IGF and the risk of IS, s-IGF-II was non-statistically significantly inversely associated with the risk of IS [28]. However, as our study is the first one investigating acute s-IGF-II levels in human stroke, our results must be considered as preliminary observations until confirmed in other IS cohorts. Regarding etiological subtypes, we observed a pronounced reduction in s-IGF-II in cardioembolic stroke compared with the controls (6.2%) and compared with the other stroke subtypes. For the other stroke subtypes, we observed higher s-IGF-II levels in cases compared to controls, although this difference was significant only in cryptogenic stroke (7.3%). The finding of lower s-IGF-II levels in cardioembolic stroke needs replication and putative mechanisms remain to be elucidated. However, IS by cardioembolic etiology partly represents another type of pathophysiology, being derived by thrombosis mainly from atrial fibrillation and cardiac arrhythmias [45], as also suggested by the proposed roles of IGF-II in cardiac epigenetics in humans [46]. Finally, the lower level of s-IGF-II in cardioembolic stroke abided after stratifying for stroke severity and CRP (data not shown), as well as remaining significant after 3 months, which argues against the notion that this finding merely reflects an acute stress response.

### 4.2. IGF-II and Mortality

The lowest quintile of acute s-IGF-II was associated with an increased risk of mortality compared to that in the four higher quintiles after adjustment for covariates. Previously, the association between s-IGF-II and mortality had not been investigated in stroke patients, and data from other study populations were limited. However, recent human research suggests that IGF-II is associated with cardiovascular risk factors. Results from genetic studies imply that the IGF-II genomic region may be involved in metabolic syndrome, type 2 diabetes, coronary heart disease, and mortality [22,23,47,48,49,50]. Interestingly, studies of the APAI polymorphism of the *IGF2* gene have demonstrated that the APAI A allele is associated with higher serum IGF-II levels and less obesity [16,20,21]. This allele was also positively associated with longevity in the Jerusalem longitudinal study, although the actual s-IGF-II levels were not presented [22]. Moreover, a decline in s-IGF-II in mid-life was associated with subsequent weight gain [38]. Furthermore, s-IGF-II in children [51] as well as in adults [52] has been negatively associated with metabolic syndrome, and IGF-II is also dysregulated in diabetic nephropathy [53]. Hence, IGF-II might participate in the regulation of obesity and metabolic syndrome, thereby indirectly being a biomarker for cerebrovascular diseases [54]. In the present study, we investigated whether s-IGF-II correlated with metabolic factors and found a modest positive relationship between acute s-IGF-II and LDL, but there was no correlation with insulin resistance as indexed by HOMA-IR. In addition, the association between low acute s-IGF-II and increased mortality was independent from cardiovascular risk factors as the association also remained significant after adjustment for these factors. Finally, low 3-month s-IGF-II was not associated with increased mortality, which further refutes that long-term effects on cardiometabolic factors were of major importance.

IGF-II is positively correlated with the risk of malignancy as well as cancer mortality [55], and IGF-II is overexpressed in cancers of different origins [56,57,58]. Overall, it is therefore unlikely that the association between low acute s-IGF-II and increased mortality in our study can be explained by an increased risk of cancer mortality.

We found an inverse correlation between acute s-IGF-II and hsCRP, suggesting a link between low s-IGF-II and systemic inflammation in the acute phase of IS. However, the significance of such interactions is uncertain as the association between the lowest quintile of acute s-IGF-II and increased risk of mortality remained after inclusion of hsCRP and stroke severity as covariates. Furthermore, in experimental IS, IGF-II was involved in inflammatory responses in activated macrophage IS [25] and provided protection from experimentally induced oxidative damage [16,17,18]. In humans, rh-IGF-II is available for research studies, but no experimental study has been carried out in the context of IS, and rh-IGF-II is not available for clinical usage. Therefore, effects of IGF-II on local inflammatory responses or oxidative stress could theoretically have been of importance. However, we cannot rule out that there are unknown confounders that we did not adjust for. Therefore, in summary, the underlying mechanisms are unclear and need to be clarified in further studies including whether IGF-II could exert direct, yet unidentified effects.

### 4.3. Modulation of IGF-II Activity

Although s-IGF-II mainly originates from the liver [3], there is also some production in other tissues including the CNS, most prominently in neurons [4,5]. Furthermore, IGF-II can pass through the blood–brain barrier [6] and potentially activate both the IGF-I receptor (IGF1R) with low affinity and the IGF-II receptor (IGF2R) with high affinity. Both are widely distributed in the brain [4,5]. Together with the six high-affinity IGF-binding proteins (IGFBPs), they are likely involved in a complex regulation of the effects of IGF-II, but to our knowledge, modulating these in terms of IGF-II signaling has not been performed. Further studies focusing on the regulation and mechanisms of how IGF-II exerts its effect in stroke may therefore benefit from measuring the actual levels of IGF-II as well as the IGFBPs in the serum as well as in the CNS.

Interestingly, there are other alternatives of modulating IGF-II activity, namely, by the so-called IGF2 mRNA binding proteins (IMPs). These exist as several subtypes and are expressed in multiple tissues including the brain, playing a major role in post-transcriptional regulation of RNAs, providing an opportunity to modulate IGF-II activity [27,59]. In adulthood, only the subtype IMP2 remains relatively highly expressed, and in genetic studies, IMP2 has been implicated in the regulation of metabolism, development, stemness, and tumorigenesis [59] and diabetes [48]. In the subgranular zone of the adult rodent dentate gyrus, the small protein RNA-binding motif protein 3 (RBM3) promoted IGF-II expression and secretion by interacting with IMP2, thereby providing neuroprotection and neurogenesis after hypoxic–ischemic brain injury [27]. It is possible to produce small compounds that stabilize RBM3, and successful, although not fully validated, attempts have been performed using a cold-inducible RNA-binding protein (CIRBP) homologue [60]. Therefore, there is a possibility that in the future, IGF-II activity could be modulated in brain disorders including stroke by targeting the IMP2-IGF-II pathway.

### 4.4. IGF-II and Functional Outcome

There was an association between the lowest quintile of acute s-IGF-II and increased risk of poor functional outcome after 3 months and 2 years, which could be in line with adverse consequences of low IGF-II in acute IS. The risk of poor functional outcome was highest after 3 months, just over 2-fold. This excess risk of poor functional outcome remained when mRS 6, i.e., dead, was left out of the analysis (data not shown), hinting that this association was not merely an effect driven by an excess mortality. However, the association between the lowest quintile of acute s-IGF-II and poor functional outcome lost statistical significance after full adjustment for covariates including stroke severity and hsCRP. Comparatively, the association between the lowest quintile and risk of mortality was more robust and also withstood our most extensive multivariate analysis in this study. This implicates that the association between s-IGF-II and functional outcome may be related to cardiovascular risk factors rather than a mechanism of neuroprotection and recovery after IS. Thus, IGF-II could be an effector rather than a primary mediator in terms of long-term functional outcome after IS.

### 4.5. Strengths and Limitations

In the context of analyzing levels of s-IGF-II, our study had a relatively large study sample including consecutive and well-characterized IS cases and population-based controls. In terms of the multivariate regression models for functional outcome and mortality, statistical power was retained with reasonable strength with the adjustment for several, but not an unlimited number of, potential confounders [61]. Although low s-IGF-II may be a pre-stroke risk marker for ischemic stroke, as shown in a primary prevention perspective [28], our analysis was confined to mortality and functional outcome from a secondary prevention perspective. We included Caucasian IS cases and controls that were below 70 years of age, which limits the generalizability of the results from a global perspective [43,62]. An advantage of our study is that the blood samples were taken twice, both in the acute phase and after 3 months. A limitation is that the acute blood samples were usually not drawn immediately after stroke onset; i.e., they were taken after a median of 4 days, hence, to some extent, reflecting the stress response in the acute phase of IS. Finally, the levels of s-IGF-II in stroke subtypes must be interpreted with caution as the number of cases in these subgroups was limited (N = 54–131).

## 5. Conclusions

Our study shows that s-IGF-II is elevated both in the acute phase and 3 months after IS. Low acute s-IGF-II was independently associated with an increased risk of mortality after IS, whereas the associations with functional outcome lost significance after adjustment for covariates. However, as our study is the first one of s-IGF-II in human stroke, our results need to be confirmed in other IS cohorts.

## Figures and Tables

**Table 1:** Baseline characteristics in ischemic stroke (IS) patients and healthy controls as well as in IS subtypes.

	Controls (n = 514)	Patients (n = 492)	Large Vessel Disease (n = 54)	Small Vessel Disease (n = 106)	Cardioembolic Stroke (n = 81) Cryptogenic Stroke (n = 131)	
Age (years)	57.2 ± 0.44	57.2 ± 0.44	58.5 ± 1.00	59.3 ± 0.66 *	58.9 ± 1.14	54.1 ± 0.94 **
Male sex, n (%)	329 (64)	315 (64)	39 (72)	66 (62)	66 (68)	77 (59)
Hypertension, n (%)	199 (39)	299 (62) ***	30 (60) ***	78 (74) ***	44 (56) **	73 (57) ***
Diabetes mellitus, n (%)	30 (6)	94 (19) ***	19 (38) ***	25 (24) ***	15 (19) ***	17 (13) ***
Current smoker, n (%)	89 (17)	193 (39) ***	27 (54) ***	46 (43) ***	29 (37) ***	50 (39) ***
hsCRP (mg/L)	3.06 ± 0.26	10.8 ± 1.00 ***	12.1 ± 2.51 ***	4.95 ± 0.66 **	16.7 ±3.18 ***	7.98 ± 1.52 **
Imputed LDL (ng/nL)	3.33 ± 0.04	3.35 ± 0.04	3.54 ± 0.15	3.48 ± 0.08	3.08 ± 0.11 *	3.32 ± 0.07
HOMA-IR	2.13 ± 0.12	4.80 ± 0.25 ***	6.14 ± 0.88 ***	5.06 ± 0.71 ***	4.59 ± 0.53 ***	3.78 ± 0.27 ***
BMI (kg/m^2^)	26.5 ± 0.18	26.7 ± 0.20	26.8 ± 0.65	27.0 ± 0.42	26.8 ± 0.51	26.4 ± 0.34
NIHSS score baseline	NA	5.32 ± 0.25	6.49 ± 0.85	3.32 ± 0.26	6.48 ± 0.78	5.24 ± 0.48
mRS score 3 months	NA	1.79 ± 0.05	2.14 ± 0.16	1.35 ± 0.10	2.01 ± 0.15	1.73 ± 0.10
mRS score 2 years	NA	1.88 ± 0.06	1.77 ± 0.24	1.49 ± 0.11	2.17 ± 0.17	1.68 ± 0.10
s-IGF-II acute (ng/mL)	712.1 ± 5.58	734.7 ± 7.08 *	747.0 ± 18.8	734.6 ± 13.7	667.9 ± 17.1 **	764.4 ± 12.2 ***
s-IGF-II 3 months (ng/mL)	NA	736.8 ± 6.91 **	751.9 ± 23.3	739.5 ± 14.8	679.4 ± 14.5 *	748.4 ± 11.0 **

Data are shown as means (±SEM) or as number (n). Values are provided for controls, the total IS cohort, and the four major etiological IS subtypes. The remaining patients had either another determined cause (n = 37) or an undetermined cause (n = 83). Differences compared to the control group were examined using ANOVA for continuous variables and the χ2-test for categorical variables (sex, hypertension, diabetes, and smoking). Furthermore, for each of the stroke etiologies, the values are compared to controls. ns, not significant. LDL, low-density lipoprotein; BMI, body mass index; hsCRP, high-sensitivity C-reactive protein; HOMA-IR, homeostasis model assessment of insulin resistance. * *p* < 0.05, ** *p* < 0.01, *** *p* < 0.001.

**Table 2:** Hazard ratios (HRs) and 95% confidence intervals (CIs) for the risk of mortality in the lowest quintile of acute serum IGF-II compared to the four higher IGF-II quintiles in the total stroke population.

Total Stroke Population	Acute Serum IGF-II Quintile 1	Quintiles 2–5	*p*-Value
Deaths, n (%)	36 (36.7%)	70 (17.9%)	
Crude	2.34 (1.56–3.49)	1.0 referent	<0.001
Multivariate model A	1.96 (1.29–2.95)	1.0 referent	0.001
Multivariate model B	1.93 (1.23–3.03)	1.0 referent	0.004
Multivariate model C	1.65 (1.04–2.63)	1.0 referent	0.035
Multivariate model D	1.64 (1.02–2.61)	1.0 referent	0.039

Hazard ratios were calculated using Cox proportional hazards regression. Data are shown as n (%) and HR (95% CI). Model A: adjustment for age and sex. Model B: age, sex, and cardiovascular risk factors (BMI, hypertension, LDL, smoking, and diabetes). Model C: age, sex, cardiovascular risk factors, and stroke severity. Model D: age, sex, cardiovascular risk factors, stroke severity, and hsCRP. LDL, low-density lipoprotein; BMI, body mass index; hsCRP, high-sensitivity C-reactive protein.

**Table 3:** Odds ratios (ORs) and 95% confidence intervals (CIs) for the risk of poor functional outcome (mRS ≥ 3) after 3 months and 2 years in the lowest quintile of acute serum IGF-II compared to the four higher IGF-II quintiles in the total stroke population.

Total Stroke Population	Quintile 1	Quintile 2–5	*p*-Value	n
**3-month mRS**				
Crude	2.30 (1.39–3.82)	1.0 referent	0.001	463
Multivariate model A	2.24 (1.33–3.79)	1.0 referent	0.002	463
Multivariate model B	1.95 (1.10–3.45)	1.0 referent	0.023	446
Multivariate model C	0.99 (0.46–2.13)	1.0 referent	0.98	446
Multivariate model D	0.71 (0.30–1.64)	1.0 referent	0.42	445
**2-year mRS**				
Crude	1.93 (1.17–3.18)	1.0 referent	0.010	485
Multivariate model A	1.76 (1.04–2.95)	1.0 referent	0.034	485
Multivariate model B	1.59 (0.90–2.80)	1.0 referent	0.11	463
Multivariate model C	0.90 (0.46–1.77)	1.0 referent	0.76	463
Multivariate model D	0.79 (0.39–1.58)	1.0 referent	0.50	462

ORs and 95% CIs were calculated using binary logistic regression. Data are shown as n and OR (95% CI). Model A: adjustment for age and sex. Model B: age, sex, and cardiovascular risk factors (BMI, hypertension, LDL, smoking, and diabetes). Model C: age, sex, cardiovascular risk factors, and stroke severity. Model D: age, sex, cardiovascular risk factors, stroke severity, and hsCRP. LDL, low-density lipoprotein; BMI, body mass index; hsCRP, high-sensitivity C-reactive protein.

## Data Availability

The data presented in this study are available on reasonable request from the corresponding author. The data are not publicly available due to legal restrictions regarding privacy and ethical issues.
